# A *sex-ratio* Meiotic Drive System in *Drosophila simulans.* II: An X-linked Distorter

**DOI:** 10.1371/journal.pbio.0050293

**Published:** 2007-11-06

**Authors:** Yun Tao, Luciana Araripe, Sarah B Kingan, Yeyan Ke, Hailian Xiao, Daniel L Hartl

**Affiliations:** 1 Department of Organismic and Evolutionary Biology, Harvard University, Cambridge, Massachusetts, United States of America; 2 Department of Biology, Emory University, Atlanta, Georgia, United States of America; Cornell University, United States of America

## Abstract

The evolution of heteromorphic sex chromosomes creates a genetic condition favoring the invasion of *sex-ratio* meiotic drive elements, resulting in the biased transmission of one sex chromosome over the other, in violation of Mendel's first law. The molecular mechanisms of *sex-ratio* meiotic drive may therefore help us to understand the evolutionary forces shaping the meiotic behavior of the sex chromosomes. Here we characterize a *sex-ratio* distorter on the X chromosome (*Dox*) in Drosophila simulans by genetic and molecular means. Intriguingly, *Dox* has very limited coding capacity. It evolved from another X-linked gene, which also evolved de nova. Through retrotransposition, *Dox* also gave rise to an autosomal suppressor, *not much yang* (*Nmy*). An RNA interference mechanism seems to be involved in the suppression of the *Dox* distorter by the *Nmy* suppressor. Double mutant males of the genotype *dox*; *nmy* are normal for both *sex-ratio* and spermatogenesis. We postulate that recurrent bouts of *sex-ratio* meiotic drive and its subsequent suppression might underlie several common features observed in the heterogametic sex, including meiotic sex chromosome inactivation and achiasmy.

## Author Summary

Mendel's first law of genetics states that two alleles of a heterozygote are transmitted to the next generation at an equal ratio. The cornerstone of population genetics, this law states that the evolutionary fate of genetic variants is solely governed by their contribution to the good of their carriers. However, meiotic drive genes—which skew transmission in their own favor—can evolve under certain circumstances, even though they cause harm to the genome as a whole. Meiotic drive elements are often enriched on the two sex chromosomes (i.e., the X and the Y) because of a lack of recombination between them. Here we describe the genetic and molecular characterization of a meiotic drive distorter on the X chromosome in Drosophila simulans. This distorter apparently formed de nova from yet another new gene. To fight back against this harmful distorter, the D. simulans genome has evolved an ingenious mechanism based on DNA sequence homology. We postulate that repeated meiotic drive invasion and its suppression could be a major mechanism for genome evolution, underlying the ultimate cause for the inactivation of sex chromosome during meiosis and the occasional loss of recombination (achiasmy), which is observed only in the heterogametic (XY) sex.

## Abstract 3

X-linked genes gain transmission advantage by killing Y-bearing sperm. The first case iof this class of rogue genes, also called *sex-ratio* distorters, has been identified in *Drosophila simulans*.

## Introduction

Sex chromosomes are believed to evolve from a pair of autosomes [1–3]. An incipient Y chromosome, like an autosome, is largely euchromatic and free to recombine, except for a small region determining sex, as exemplified by species such as the papaya plant [4] and the medaka and stickleback fish [5,6]. On an evolutionary time scale, the nonrecombining region of the Y will generally expand to include most or all of the chromosome, accompanied by an accumulation of transposable elements and other repetitive sequence, as well as mutational inactivation of most of the protein-coding genes. Only a small number of genes remain active in a mature Y chromosome, such as that in humans or *Drosophila*. Some Y-linked genes are vestiges of the degeneration process, while others have originated from autosomes as a result of recruiting male-specific genes such as those that function in spermatogenesis [7–10]. Accompanying the evolution of sex chromosomes, at least two problems of biological significance arise. One problem is the unequal gene dosage of sex-linked genes between the XY sex and the XX sex. Because of Y degeneration, most genes on the X have only one active copy in the XY sex but two in the XX sex. Myriad strategies to compensate the dosage inequality have been exploited by various species, and some of these mechanisms are now understood in molecular detail in model organisms of fly, worm, and mouse [11].

Another substantial but less obvious problem consists of genetic conflicts over the sex ratio among various parts of a genome, which would allow optimal transmission of their own genes. A corollary to sexual reproduction is Fisher's well-known principle that the sex ratio must be equal for a panmictic population of dioecious species [12]. However, as noted long ago, Fisher's principle applies only to autosomal genes but not to sex-linked genes. Genes linked to one or the other sex chromosome would have a selective advantage were the sex ratio in the population skewed [13]. Because of the genetic isolation between the sex chromosomes, mutations biasing the sex ratio can easily accumulate and enhance each other as long as their deleterious effects are offset by their biased transmission. Thus, the evolution of sex chromosomes leads to an intrinsic conflict among the X, the Y, and the autosomes with regard to sex ratio.

Many cases of sex ratio distortion (*sex-ratio* hereafter) have been documented, particularly in taxa where intensive laboratory investigation is possible [14]. Because of the biased sex ratio, suppressors unlinked to a distorter are strongly selected to restore the Fisherian sex ratio [15]. The occurrence of *sex-ratio* in a population can often be transient and easily escape notice. However, recurrent bouts of *sex-ratio* invasion and suppression can modify the genetic architecture of gametogenesis to such an extent that hybrid incompatibility can be driven to evolve among isolated populations. In other words, genetic conflicts can be a key mechanism for speciation [16–18].

Several cases of *sex-ratio* have been reported in D. simulans [19–23]. In a companion paper, we reported the cloning of an autosomal *sex-ratio* suppressor [24]. As Fisher's principle predicts, there must exist an X-linked *sex-ratio* distorter to which this suppressor corresponds. Here we report the characterization of such a distorter. More generally, we speculate that *sex-ratio* distortion might underlie the evolution of meiotic sex chromosome inactivation and achiasmatic meiosis, two biological phenomena whose evolutionary origins still remain mysterious.

## Results

### A *sex-ratio* Distorter on the X Chromosome (*Dox*)

We previously cloned a D. simulans gene, *not much yang* (*nmy*, polytene chromosome position 87F3), in which the homozygous male mutant displays a female-biased sex ratio. This gene belongs to the Winters *sex-ratio* system, one of three independent *sex-ratio* systems found in this species [24]. We inferred that the wild-type (*Nmy*) function is a suppressor of *sex-ratio* distortion, and that there must be a corresponding X-linked *sex-ratio* distorter according to Fisher's principle of sex ratio evolution. By happenstance, we found an X chromosome that did not express the *sex-ratio* phenotype in homozygous *nmy* males (Figure S1). This X chromosome was thought to have a loss-of-function mutation in the gene(s) causing *sex-ratio*. We designate the mutant gene as *distorter on the X* (*dox*). Other X chromosomes, including one from the stock *y w^am^ v^2^ f^66^*, did express *sex-ratio* when tested in the *nmy* background and were postulated to carry the distorting allele *Dox* (Figure S2).

**Figure 1:**
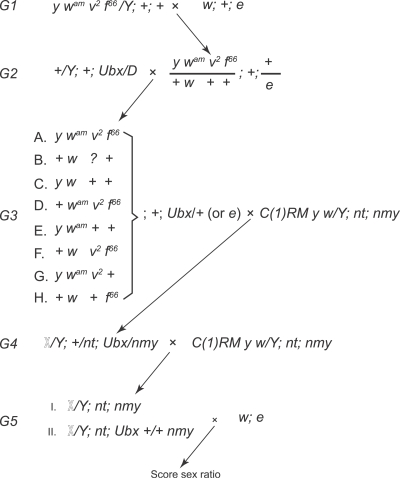
Cross Scheme for Mapping *dox* One of the parental X chromosomes (*y w^am^ v^2^ f ^66^*) has functional distorter(s), whereas the other (*w*) does not. Single-male matings were set up for all X chromosome recombinants in *G3*. All recombinants, represented by X in *G4* and *G5*, were tested for *sex-ratio* in an isogenic background *nt; nmy* in *G5* (I) as well as in a control background of *nt; +/nmy* (II). Two classes of recombinants (F and H) with the same visible phenotype were distinguished by progeny from additional crosses with *y w^am^ v^2^ f ^66^* females in *G5*. However, class B (*+ w ? +*) may be a mixture of *+ w + +* and *+ w v^2^ +*, which were not distinguished.

**Figure 2:**
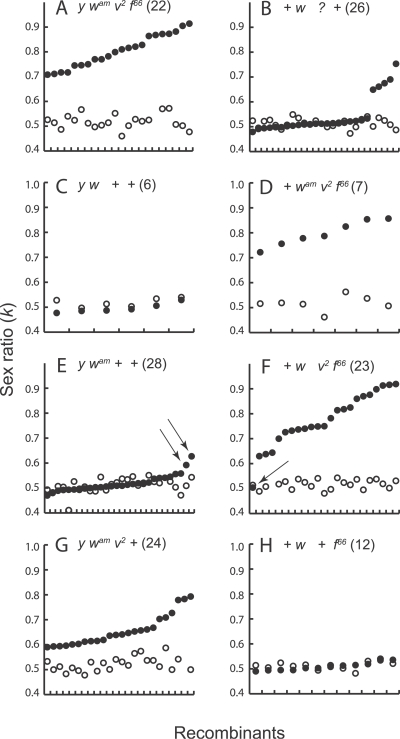
Sex Ratios (*k*) Scored for the Preliminary Mapping Each recombinant X chromosome in classes A–H (Figure 1) was tested against *nmy* (filled circle) and its sex ratio was rank-ordered within each class. Also shown are the corresponding sex ratios tested against *nmy*/*+* (open circles) as control and the number of recombinants tested for each class (in parenthesis). Three exceptional recombinants are indicated with arrows.

A preliminary mapping of *dox* was carried out through the scheme described in Figure 1. Recombinant X chromosomes were tested for sex ratio (proportion of females or *k*) in the *nmy* background (I in G5 of Figure 1) as well as in the *nmy/+* background as control (II in G5). A total of 148 X chromosomes tested could be grouped into eight genotypic classes (Figure 2A–2H). Several inferences can be drawn from the results. First, there are two and one recombinants in classes E and F, respectively, which are exceptional and thus informative in the mapping of *dox*, allowing it to be placed closely proximal to *v* at a distance of about 6% (3/51) of the *w–v* interval. Second, the major *sex-ratio* distorter shows less strength of distortion when a gene in the vicinity of *f* is absent. The reduced distortion can be inferred by comparing classes A (*k* ± standard error of the mean [SEM] = 0.803 ± 0.014, *n* = 22), D (0.797 ± 0.019, *n* = 7), and F (0.786 ± 0.020, *n* = 22, excluding one losing the major distorter) with class G that has a significantly lower sex ratio of 0.655 ± 0.013 (*n* = 24) (*t*-test, *p* << 0.001). The first three classes have similar sex ratios (analysis of variance [ANOVA], *p* = 0.748). We call the gene near *f* an *enhancer of Dox* (*E*(*Dox*)) because it alone does not cause sex ratio distortion (class H, 0.509 ± 0.004). In light of the above reasoning, the five class B recombinants showing *sex-ratio* probably have an inferred genotype of *w Dox v^2^ e(Dox)* (0.686 ± 0.018), the same as class G with respect to *sex-ratio* distorter and enhancer (*t*-test, *p* = 0.157). Finally, based on numerous SNP sites found between the two parental X chromosomes, we genotyped a selected subset of the 148 chromosomes and narrowed the location of *dox* to a region of 215 kb between *CG15316* (8E1–4) and *nej* (8F7–9), which falls within an interval defined by two visible markers *lz* and *v*.

### Fine Mapping of *dox*

**Figure 3:**
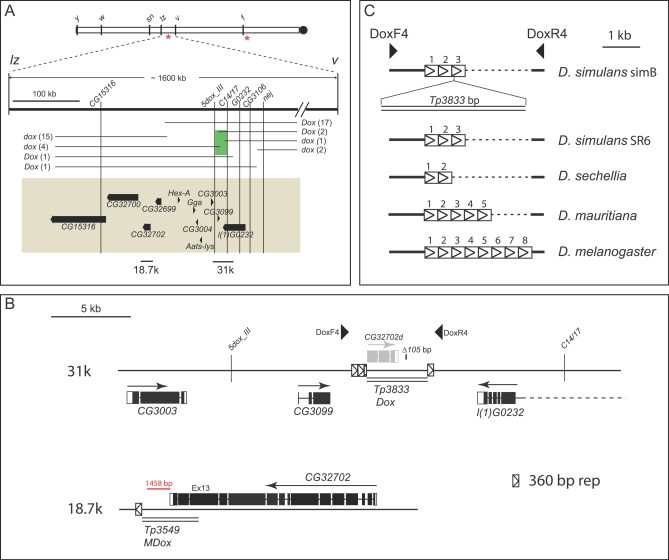
Fine Mapping and Positional Cloning of *dox* (A) The crossover intervals and the *sex-ratio* phenotype of the definitive 43 recombinants between *lz* and *v*. *dox*: normal sex ratio; *Dox*: sex ratio distorted. The number of similar recombinants is included in parenthesis. The seven most informative recombinant events fall within the interval *5dox_III* and *C14/17* (green region). Some of the annotated genes in D. melanogaster in this region are also shown (yellow region) (http://www.flybase.org/cgi-bin/gbrowse/dmel/). The two regions marked as 18.7k and 31k in the D. simulans simB strain have been sequenced from PCR products and phage clones. * represents *Dox* at left and *E*(*Dox*) at right. (B) Annotations of the 18.7k and 31k regions in D. simulans. Genes orthologous to D. melanogster are shown (arrow: orientation of transcripts; line: introns; open box: untranslated region of mRNA; filled box: coding sequence). A transposition of 3,833 bp (double lines, *Tp3833*) in the *Dox* region can be recognized as originating from the downstream region of *CG32702* (*Tp3549*). The positions of the 105-bp deletion (Δ105) and the exons of *CG32702* within *Tp3833* (*CG32702d* in gray) are shown. A repetitive sequence with a 360-bp consensus has also been recognized in both regions. Primers DoxF4 and DoxR4 were used to amplify this region for cross-species comparisons. In the *Tp3549* region, a sequence of 1,458 bp (red line) has no homolog in *D. melanogaster CG32702*. (C) Comparison of the *Dox* region among several species of the D. melanogaster subgroup. Sequences from eight strains of D. simulans, and one strain each from D. sechellia (3588), D. mauritiana (w12), and D. melanogaster (*y; cn bw sp*) were compared. *Tp3833* is present in seven strains of D. simulans (represented by simB) but is absent in *SR6*. It is also absent in all other species examined.

The fine mapping of *dox* began with the construction of two X chromosomes of *lz^s^ Dox v^2^ f^66^* and *y w^am^ dox*, whose phenotype with regard to *Dox* was confirmed by testing in a homozygous *nmy* background through the scheme described in Figure 1 (G3 through G5). A cross of + + *lz^s^ Dox v^2^ f^66^*/*y w^am^ + dox + +* females to *Ubx*/*D* males was set up, and 324 recombinants with crossovers between *lz* and *v* were obtained. We picked 22 *lz^s^* and 21 *y w^am^ v^2^ f^66^* X chromosomes with crossovers falling between *CG15316* and *nej* to further test their *sex-ratio* phenotype in a homozygous *nmy* background, again using the scheme in Figure 1 (G3 – G5) (Figure 3A). The cross in G4 was carried out at 18 °C so that the *sex-ratio* phenotype of *Dox* can be fully expressed in G5 [24]. Each of the 43 recombinants was unambiguously classified as either *Dox* or *dox*.

Four SNP markers were found in the *CG15316–nej* region (Table S1), and these were used to demarcate the crossover points for the 43 recombinants. There are two *Dox* and five *dox* lines, with their crossovers falling between the markers *5dox_III* and *C14/C17*. We sequenced ∼31 kb embracing this region (Figure 3A). The two parental alleles are identical for the 20,791 bp within the *5dox_III–C14/C17* interval, except for a deletion of 105 bp (Δ105) in *dox* (Figure 3B). We confirmed the predicted presence or absence of the Δ105 element in the final seven informative recombinants.

We sequenced six other D. simulans strains in the region between the primer pair DoxF4-DoxR4 that spans the Δ105 sequence (Figure 3B; Text S3). Two types of haplotypes were recognized. One is from the *SR6* X chromosome that carries the Paris *sex-ratio* distorters [24,25]. Three copies of a 360-bp repeat were found within this haplotype. The other type is shared by all the other strains, with an insert of 3,833 bp found within the last 360-bp repeat.

This 3,833-bp fragment has sequences homologous to the last three exons from the gene *CG32702* of D. melanogaster. The *CG32702* ortholog is missing in the current annotation of D. simulans genome (Release 1.0, http://genome.ucsc.edu/). However, we did obtain a sequence of 18.7 kb covering the orthologous *CG32702* region in D. simulans as well as its full length cDNA of 11,550 bp (Figure 3B and Text S1). The transcript consists of 15 exons, largely agreeing with the computational annotation of this gene in D. melanogaster, except for differences in two splice sites and one extra exon at the 5′ end.

Apparently, this 3,833-bp insert (designated *Tp3833*) was duplicated and transposed from a sequence of 3,549 bp (designated *Tp3549*) in the 3′ region of *CG32702*. Note that one copy of the 360-bp repeat is also present next to *Tp3549*, suggesting that this repeat may have facilitated the transposition. The last two exons and part of exon 13 (Ex13) of *CG32702* are still intact in the *Tp3833* region (*CG32702d*, Figure 3B).

Sequences from the homologous region between DoxF4-DoxR4 were obtained from one strain of each of the sibling species D. sechellia, D. mauritiana, as well as D. melanogaster. All species resemble *SR6* in having various copy numbers of the 360-bp repeat (Figure 3C). A phylogenetic analysis of these 360-bp repeats shows a monophyly of the eight copies from D. melanogaster, but a reticulate relationship among the rest, suggesting shared evolutionary history of this intergenic region in the D. simulans clade (Figure S3). However, it remains to be determined whether a *Tp3833*-like sequence can be found in D. mauritiana. The existence of a functional *Nmy* strongly suggests that a corresponding *sex-ratio* distorter like *Dox* may still segregating in this species [24].

Within *Tp3549*, a fragment of 1,458 bp replaces a fragment of 1,408 bp downstream of the 3′ end of *CG32702* in *D. melanogaster,* and these two sequences have no homology (Figure 3B). Database searches suggest that the 1,458-bp sequence is absent from D. sechellia, *D. yakuba*, and *D. erecta*, but some similar fragments of 300–600 bp can be found dispersed in these genomes, often in multiple copies. Transcripts within *Tp3549* were detected, and a new gene, which we designate as *Mother of Dox* (*MDox*), is defined (see below). *Tp3549* and *Tp3833* thus represent a fluid portion of the *Drosophila* genome that occasionally gains new functions.

### Gene Structure of *Dox*

**Figure 4:**
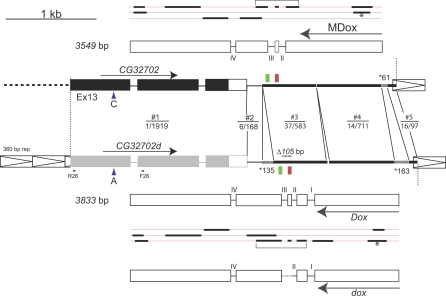
Molecular Structure of *Dox* and *MDox* Both *Dox* and *MDox* have transcripts in the opposite direction of *CG32702d* or *CG32702*, respectively. The genomic sequences of *Tp3833* (*Dox*) and *Tp3549* (*MDox*) are largely homologous except for one indel of 135 bp (*135) and another short sequence (*61 in *MDox* versus *163 in *Dox*). The 360-bp repetitive sequence may have been involved in the transposition event of *Tp3833*. Five homologous segments of sequences are recognized. The nucleotide divergence is shown for each of them (#1–#5) expressed as nucleotide difference over segment length. Segment #1 corresponds to the last exons of *CG32702* and it has only one nucleotide substitution (A versus C in Ex 13) between these two paralogs. R26 and F26 are the two PCR primers used to amplify genomic DNA and cDNA in this region (see Figure S4). The transcripts of *Dox* and *MDox* were determined by RT-PCR, 5′- and 3′-RACE with primers that can distinguish between these two genes. The intron–exon boundaries of these two genes are largely the same between *Dox* and *Mdox*, so they are annotated together. However, the 91-bp intron I of *Dox* has never been found from *MDox*. Several alternative splicing forms including earlier termination have been found. In some RT-PCR products, introns III and IV of *MDox* are not spliced out. Other splice forms may yet be uncovered. The 105-bp deletion (dashed line) in *dox* causes the loss of exon III in the otherwise identical transcripts as compared to *Dox*. Identical tandem repeats of a 42-bp element (green and red rectangles) are present in both *Dox* and *MDox* transcripts. It is unclear which ORF, if any, is actually translated in each transcript. All potential ORFs larger than 100 bp are shown for all three reading frames (thick black lines on thin red lines). Two ORFs of 157 and 107 aa across the exon III region in the *Dox* and *MDox* transcripts, respectively, are marked with brackets and are the strongest candidate for translation (see Text S2). The best ORF predicted by GENSCAN are marked with an asterisk (http://genes.mit.edu/GENSCAN.html).

Initially the truncated version of *CG32702* (*CG32702d*) appeared to be the best candidate for *Dox* because of its perfectly conserved open reading frames (ORFs) and intron–exon boundaries (Figure 3B). However, we have not detected transcripts from *CG32702d*. Extensive 5′- rapid amplification of cDNA ends (RACE) experiments using gene-specific primers targeting *CG32702d* all failed. A 3′-RACE experiment did recover cDNAs, but they could be transcribed from the 3′-end of *CG32702*, not of *CG32702d*. There is a divergent site (C/A) between *Tp3549* and *Tp3833* in the 1,919-bp region corresponding to the last three exons of *CG32702*. Using the primers CG32702seqF26 and CG32702seqR26 (F26 and R26 in Figure 4; Text S3), only the *CG32702* sequence can be amplified from cDNA (Figure S4). *CG32702d* is therefore unlikely to be transcribed or its expression is too low to be detected by reverse-transcription PCR (RT-PCR).

On the other hand, we have detected transcripts that cover the region of Δ105 in the opposite direction of *CG32702d* (Figure 4). Two transcripts from the allele *Dox* were recovered with either four or three introns. Their full lengths are 2,781 bp and 2,690 bp, respectively. From the allele *dox*, we have also recovered two full-length cDNAs identical to those of *Dox*, except that the exon III, 42 bp in length, is missing because of the deletion Δ105 (Figure 4). This 42-bp element is tandemly repeated in the cDNA of *Dox* but has only one copy in that of *dox*. Within *Tp3549*, we have also recovered a full length cDNA antisense (2,564 bp) to the 3′ end of *CG32702* (Figure 4). *MDox* like *Dox* also has three introns in exactly the same sites, as well as the tandem repeats of 42 bp present in its cDNA (Figure 4).

Surprisingly, all transcripts from the *Dox* and *MDox* loci have very limited coding potential. The largest ORFs of *MDox* in all three frames are shown in Figure 4, and all but one fail to match any known sequences by BLASTX searches through the *nr* database. The one ORF that was predicted by Genscan encodes 62 amino acids (aa), and this ORF highly matches (57/62 or 91% identity) the C terminus of a D. melanogaster gene named *CG8664* (located in region 15F7, proximal to the gene *f*). Similarly, only one of the largest ORFs of *Dox* has BLASTX hits. This is again the ORF of 69 aa predicted by Genscan and is homologous to the 62 aa ORF of *MDox*, although only part of it matches to *CG8664* (38/44 or 86% identity) due to a frameshift mutation. *CG8664* has no known biological functions or phenotypes. In the orthologous position of *CG8664* in the current D. simulans genome annotation, a fragment of 2,084 bp, instead of a *CG8664* homolog, has been found. Part of this 2,084-bp fragment, approximately equivalent to the 1,458-bp element mentioned above (Figure 3B), is recognized and has a high similarity (99.3% identity) to a region within *Dox*. If the existence of this partial paralog of *Dox* in the *f* region is confirmed by experiment, it would be interesting to test it as the candidate gene for *E(Dox)*.

The pair of tandem 42-bp elements essential for a functional *Dox* are located within an ORF of 157 aa (Figure 4). A 14-aa domain encoded by this 42-bp element has no known functions. If this ORF is ever translated, the tandem 14-aa domains appear to be required for the wild-type function of *Dox* as a *sex-ratio* distorter. Coding or noncoding, the molecular mechanism underlying the effect of *Dox* in rendering Y-bearing sperm dysfunctional awaits further experimental investigation. The wild-type function of *MDox* is not known, although the presence of the critical tandem repeats of the 42-bp element suggests its biochemical similarity to *Dox*.

### *Nmy* Suppresses *Dox* through Homology Effect

**Figure 5:**
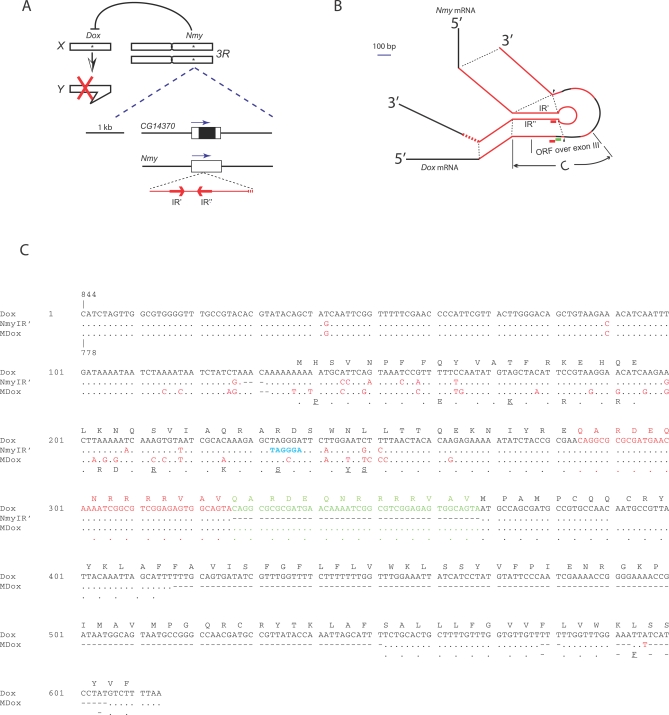
Homology Among *Dox*, *Mdox*, and *Nmy* (A) Schematic of the Winters *sex-ratio* system. An autosomal suppressor, *Nmy*, is apparently a new gene created by a 2,041-bp insertion (red) in the gene *CG14370*. The insert contains a pair of almost perfect IRs of 345 bp (red arrows, IR' and IR'') and they are required for the suppression of *Dox*. *Nmy* originated through a retrotransposition event from *Dox*, because all of the 2,041 bp consists of paralogous sequence from cDNA of *Dox* (red line) and the 5′ region upstream of *Dox* transcription (dashed red line) [24]. (B) A detailed comparison between transcripts of *Dox* and *Nmy*. Paralogous sequences are in red. The 5′ and 3′ ends of *Nmy* (black line) are from *CG14370*. IR' might have duplicated from IR” after the retroposition [24], while some sequences (black between the two small arrow heads) in *Dox* no longer have paralogs in *Nmy*. The 42-bp elements are shown in red and green color, as in Figure 4. The *dox* allele has lost one of the 42-bp elements in the transcript. A dsRNA stem is presumably formed between the two IR's. The critical region marked as “C” is detailed in (C). (C) The critical region “C” with a base-by-base comparison among *Dox*, *Nmy*, and *MDox*. Identical bases or amino acids are represented by a period, and deletion by a dash, and divergent bases are in red. This region starts at position 844 (778) of the *Dox* (*MDox*) transcript and the beginning of IR', respectively. IR' and IR'' are identical except for a 6-bp (TAGGGA) deletion in IR' (cyan). The two tandem 42-bp elements are also shown in red and green, respectively on the *Dox* sequence. The amino acids encoded by the ORF in this region are shown in the top (*Dox*) and bottom lines (*MDox*), respectively. These two ORFs have an amino acid identity of 94/107 (88%) and similarity of 100/107 (93%) (underlined).

The phenotype of *Dox* as a *sex-ratio* distorter is uncovered if its suppressor, *Nmy*, is nonfunctional (Figure 5A) [24]. The *Nmy* transcript appears to form a stem-loop structure with a double-stranded RNA (dsRNA) stem of 345 bp, and small interfering RNAs (siRNAs) produced from the dsRNA stem could target and suppress *Dox* [24]. Hence, homology between *Dox* and *Nmy* is anticipated. Indeed, sequence comparisons suggest that *Nmy* originated from *Dox* through a retrotransposition event [24]. Specifically, the 345-bp dsRNA sequence from *Nmy* has extensive homology to the potential ORF of both *Dox* and *MDox* that contains exon III (Figures 5B). The critical 42-bp element falls within an 85-bp region that has a perfect match with the stem region (positions 264–390 in the alignment of Figure 5C). Whether or how either *Dox* or *MDox* is regulated by these hypothetical siRNAs is currently under investigation.

**Figure 6:**
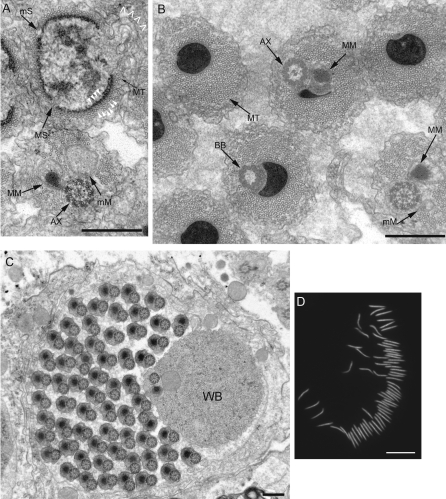
Spermatogenesis of Double-Mutant *dox; nmy* Is Normal (A–C) TEM images of sperm maturation. (A) Early post elongation stage of a spermatid head (upper) and tail (lower). Normal nuclear condensation can be seen apposed to rows of microtubules (white arrowheads), while nucleoplasm is eliminated (v). (B) Late post elongation stage of spermatid heads with homogeneously condensed nuclei (upper left and right). Also note the normal head-tail alignment (middle) and the tail (lower right). (C) Post individualization stage of a sperm bundle with 61 normal tails and one degenerated tail in the waste bag (WB). (D) DAPI images of 64 spermatid heads in a bundle at the stage of post elongation. Abbreviations used in annotation: AX (axoneme); BB (basal body); mM (minor mitochondrial derivative); MM (major mitochondrial derivative); mS (minor strip); MS (major strip); MT (microtubule); WB (waste bag). Scale Bars: 500 nm (A–C); 20 μm (D).

The possibility that *Dox* evolved solely as a *sex-ratio* distorter and for no other reasons is supported by the normal phenotype of the double mutant *dox; nmy*. We have shown previously that the etiology of the Winters *sex-ratio* is the degeneration of the Y-bearing spermatids during their maturation, as observed both through transmission electron microscopy (TEM) and through light microscopy [24]. We carried out similar observations of the spermatogenesis of *dox; nmy* males at 16 °C. All stages of spermatid maturation appear to be normal as also found in *Dox; Nmy* wild type (Figure 6A–6D, in comparison to Figures 4 and 5 in [24]). Quantitatively, 5.7% (*n* = 1108) of spermatid heads appear to be abnormal under TEM, in a proportion similar to wild-type *Dox*; *Nmy* (5.8%, *n* = 1903). With 4',6-diamidino-2-phenylindole (DAPI) staining, no abnormal spermatid head was observed among the 1,416 heads examined. Consistent with these cytological observations, the sex ratio of progeny from the *dox; nmy* males at 16 °C was 54%. As a comparison, a *dox; Nmy* male was similarly examined. No abnormal heads were observed among 1,058 spermatids, and the sex ratio when tested was also 54%. All the evidence together suggests that *Dox* is not an essential gene and is fully dispensable. *Nmy* is also dispensable if *Dox* is absent.

### Evolution of *sex-ratio* in D. simulans

The fate of a *sex-ratio* system can be loss, fixation, or stable polymorphism. Apparently, the Winters *sex-ratio* is still segregating in D. simulans [24]. The same is true for the Paris *sex-ratio* system that has been found in the same species [21]. Evidence from molecular population genetics shows that the Paris *SR6* X chromosome has swept through African and Indian Ocean islands only recently (less than 20 thousand years ago [ka]) [25]. The presence of a functional *Nmy* suppressor in D. mauritiana suggests that the Winters *sex-ratio* evolved in the ancestor of the D. simulans clade [24]. The following genetic evidence will enforce the above conclusions and help to compare the evolutionary history of these two *sex-ratio* systems.

**Figure 7:**
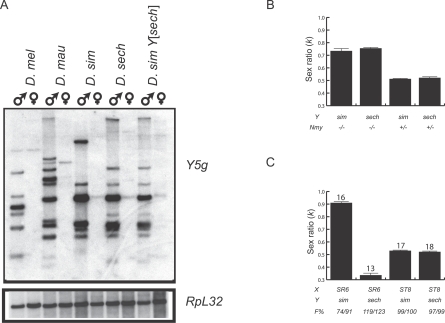
The Y Chromosome from D. sechellia Is Sensitive to *Dox* but Distorts *SR6*, Resulting in a Male-Biased Sex Ratio (A) Fingerprinting the Y chromosomes from D. melanogaster sibling species. Genomic DNA was cut with *Hind* III and probed sequentially with the probes *Y5g* and *RpL32*. The probe *Y5g* is composed of sequences from five known Y-specific genes (Figure S6). *RpL32* is an autosomal gene at 99D3. Stocks used: D. mel: *D. melanogaster Canton-S*; D. mau: *D. mauritiana w12*; *D. sim: D. simulans* simB; *D. sech: D. sechellia 3588*; *D. sim Y*[*sech*]: isogenic to simB (*w; nt; Nmy*) except that the *Y* is from *D. sechellia 3588*, as confirmed here. (B) Test of the sensitivity of *Y*[*sech*] to *Dox*. *Dox*/*Y*[*sim*] or *Dox*/*Y*[*sech*] males in the background of *nmy* or *nmy*/*Nmy* were constructed by the following scheme: simB or *D. sim Y*[*sech*] males were crossed to *P*40L12 (*w*; *nt*; *P*40L12 *nmy*/*Nmy*] or *P*40B13 (*w*; *nt*; *P*38B13 *Nmy*/*Nmy*) females. Ten sublines of each combination were set up by mating single F1 male (*P*40/*Nmy*) to SR1227 (*w*; *nt*; *nmy*) females. The following four genotypes of males were obtained in the F2: (1) *Dox*/*Y*[*sim*]; *nmy* (*w Dox*/*Y*[*sim*]; *nt*; *P*40L12 *nmy*/*nmy*). (2) *Dox*/*Y*[*sech*]; *nmy* (*w Dox*/*Y*[*sech*]; *nt*; *P*40L12 *nmy*/*nmy*). (3) *Dox*/*Y*[*sim*]; *Nmy*/*nmy* (*w Dox*/*Y*[*sim*]; *nt*; *P*40B13 *Nmy*/*nmy*). (4) *Dox*/*Y*[*sech*]; *Nmy*/*nmy* (*w Dox*/*Y*[*sech*]; *nt*; *P*40B13 *Nmy*/*nmy*). Three males of each subline were tested for *sex-ratio* at room temperature. *Dox; nmy* males express s*ex-ratio* regardless of the origin of the Y chromosome. *Y*[*sech*] and *Y*[*sim*] are similar in their sensitivity to the sex ratio distortion effect of *Dox* (*t*-test, *p* = 0.341). (C) Test the sensitivity of *Y*[*sech*] to *SR6.* The X chromosome *SR6* carries the Paris *sex-ratio* distorters, while *ST8* is the standard nondriving chromosome. Four genotypes of *SR6*/*Y*[*sech*] and its controls were constructed by crossing F1 females obtained from the crosses simB or *D. sim Y*[*sech*] x *C(1)RM yw; nt; Nmy* to *SR6* or *ST8* males: (1) *SR6*/*Y*[*sech*] (*SR6*/*Y*[*sech*], *nt*/*+*; *Nmy*); (2) *SR6*/*Y*[*sim*] (*SR6*/*Y*[*sim*], *nt*/+; *Nmy*); (3) *ST8*/*Y*[*sech*] (*ST8*/*Y*[*sech*], *nt*/+; *Nmy*); and (4) *ST8*/*Y*[*sim*] (*ST8*/*Y*[*sim*], *nt*/+; *Nmy*). Individual males of the above four genotypes were tested for *sex-ratio* by crossing to three *w; e* females (column ± SEM, with sample size). *SR6* distorts *Y*[*sim*] as reported before [81], and *Nmy* does not suppress *SR6*. Unexpectedly, *Y*[*sech*] actually distorts *SR6*. *F%*: F3 males obtained in the above *sex-ratio* test matings were crossed singly again to three *w; e* females.

We have introgressed the Y chromosome of D. sechellia into D. simulans (*D. sim Y*[*sech*]) in a background isogenic to simB (Figure S6). The success of this introgression was confirmed by fingerprinting with a Y-specific probe *Y5g* (Figure 7A and Figure S2). The D. sechellia Y chromosome was thus tested against the driving effect of either *Dox* (Winters) or *SR6* (Paris). The *Dox/Y*[*sech*] male expresses *sex-ratio* if *nmy* is homozygous, but does not if one copy of the functional *Nmy* gene is present (Figure 7B). Hence the D. sechellia Y chromosome is equally sensitive to *Dox* as is the D. simulans Y chromosome. Intriguingly, *SR6*/*Y*[*sech*] males exhibit male-biased sex ratio distortion (*k* = 0.33). Unfortunately, similar introgression of the Y chromosome from D. mauritiana cannot be made because *D. sim Y*[*mau*] is sterile [26].

The above observations are consistent with the earlier estimate that the origin of *Dox* predates speciation among D. simulans, D. mauritiana, and D. sechellia about 200–400 ka [27], whereas *SR6* arose in D. simulans after the speciation [24,25]. Assuming that an Y-linked distorter causing male-biased sex ratio distortion has little chance of persistence as compared to an X-linked one causing female-biased sex ratio distortion [13,28], we suppose that the *Y*[*sech*] still bears sensitive sequence to *Dox* as in the ancestral Y of the three species. We suggest that the male-biased sex ratio expressed by *SR6*/*Y*[*sech*] is a sign of evolutionary independence between the *Y*[*sech*] and the *SR6* distorters.

The etiology of *SR6* has been attributed to loss or breakage of the D. simulans Y chromosome during meiosis II in *SR6*/*Y*[*sim*] males [29,30]. Our results support earlier findings by showing that male progeny from the *SR6*/*Y*[*sim*] father are sterile at a frequency of 19%. However, the frequency of sterile male progeny from an *SR6*/*Y*[*sech*] father is only 3% (Fisher's exact test, *p* < 0.0001), a number that is not different from the control (2%, Fisher's exact test, *p* = 0.284) (Figure 7C). It is possible that *SR6* does not cause loss or breakage of the *Y*[*sech*], hence the etiology of the male-biased sex ratio may be different from similar *male sex-ratio* (*msr*) cases reported in D. pseudoobscura [31] and in D. affinis [32], where a large number of nullo XY sperm are produced. The unique cytological mechanism underlying the male-biased sex ratio in *SR6*/*Y*[*sech*] males again suggests that the unequal sex ratio is a neomorph created by a genetic incompatibility between the two chromosomes, rather than a shared evolutionary history of *sex-ratio*.

## Discussion

A *sex-ratio* meiotic drive distorter, *Dox*, has been identified. *Dox* is a new gene that arose from yet another new gene *MDox*. Intriguingly, both *MDox* and *Dox* appear to be transcribed as noncoding RNAs or as mRNAs with very limited coding potential. *Dox* was also the precursor for the origin of the autosomal suppressor *Nmy* by a retrotransposition process. *Dox* functions solely as a *sex-ratio* distorter and is not essential, because the mutant *dox* males have normal sex ratio and spermatogenesis. The *Dox*/*Nmy* system is the first that has been characterized at the molecular level for *sex-ratio* meiotic drive, a widespread biological phenomenon that is promoted by the evolution of heteromorphic sex chromosomes.

### *Nmy* Suppresses *Dox*, Possibly through an RNAi Mechanism

The gene structures of *Dox* and *Nmy* strongly suggest that an RNAi mechanism is involved, just as in numerous transgenic studies where inverted repeats (IR) were used to silence target genes in eukaryotes (e.g., [33]). Most likely, the suppression of *Dox* by *Nmy* is through a classic RNAi pathway, also known as post-transcriptional gene silencing (PTGS), which has been under intensive genetic and biochemical studies (reviewed in [34]). In essence, 21–23 nucleotide (nt) siRNAs processed from dsRNA are responsible for guiding the active RNA-induced silencing complex (RISC) to homologous mRNA, resulting in the latter's subsequent cleavage [35]. The PTGS model for *Dox*/*Nmy* interactions can be readily tested by comparing the steady-state mRNA levels of *Dox* between *Dox; Nmy* and *Dox; nmy* males, and by detecting the binding of specific siRNAs with the RISC components. Because PTGS happens in the cytoplasm, and spermatid nuclei within a cyst share the same cytoplamic syncytium, the final gene product of *Dox* likely has a localized deleterious effect in the Y-bearing spermatid nuclei, whereas the presence of a Y or absence of an X must provide the primary cue that eventually leads to abnormal maturation of the Y-bearing sperm heads.

Alternatively, a different type of RNAi mechanism could be involved in the *Dox*/*Nmy* interaction. A class of small RNA in the size range 24–29 nt has been identified as silencing intermediates in the control of repetitive sequences such as retrotransposons [36]. Unlike the classic RNAi machinery, the core proteins do not require DCL-1, DCL-2 and AGO2, and a different type of RNAi pathway (repeat-associated small interfering RNAs or rasiRNAs) has been proposed [36]. The rasiRNA pathway has also been shown to be responsible for silencing a possible cryptic *sex-ratio* meiotic drive distorter, *Ste*, in D. melanogaster [37]. The Y-linked *Su*(*Ste*) suppresses the deleterious effects of the X-linked *Ste*, including male sterility and meiotic drive [38]. Both *Ste* and *Su*(*Ste*) consist of repeats that share extensive homology, and rasiRNAs were shown to be the information carrier for the target specificity [36,39,40].

A third possible mechanism for silencing *Dox* might be at transcriptional level in a manner of co-suppression as first observed in plant transgenics, where the expressions of both an endogenous gene and the homologous transgene were down-regulated [41]. This type of transcriptional gene silencing (TGS) has been demonstrated in *Drosophila* [42,43], and it requires physical contacts between homologous sequences and Polycomb group (PcG) proteins [44]. Note that an intact pair of inverted repeats is not required for an efficient TGS (e.g.,[44]). In our case, *nmy*[1427] is a loss-of-function mutation that does not have an intact pair of inverted repeats but does have a 1.2-kb sequence paralogous to *Dox,* arguing against this type of TGS as a strong candidate mechanism for silencing *Dox* [24].

### Evolutionary Cause for Meiotic Sex Chromosome Inactivation and Achiasmatic Meiosis: The Drive Hypothesis

The X chromosomes of many species are condensed precociously in prophase of meiosis I when active transcription peaks in the autosomes [45]. The existence of meiotic sex chromosome inactivation (MSCI, also known as X chromosome allocycly) has been well established in several model organisms, either directly through the observation of precocious heterochromatin sex bodies [46–48], or indirectly from genetic analysis of X-autosome translocations [49] as well as with genome-wide gene expression studies [50–55]. Recently, MSCI has been demonstrated in D. melanogaster by assaying transgenic expressions in the X chromosome [56]. Though sex bodies are the direct evidence for MSCI, they have not been observed in most species examined so far, including *Drosophila* [57]. The status of MSCI may be assayed with more sensitive methods such as the detection of histone modifications that relate to transcriptional activity (e.g., [48]).

There are several hypotheses for the evolution of MSCI. One hypothesis is that MSCI evolves because of a need to suppress recombination between the two sex chromosomes [58]. Another hypothesis was coined as the SAXI hypothesis (sexual antagonism and X inactivation). Because the X spends 2/3 of its evolutionary history in females, the X will be depleted of male-specific genes, and a feminized X would be under selection to be silenced during male meiosis [59]. A third hypothesis has been suggested in the light of the discovery of the meiotic silencing of unpaired DNA (MSUD) in Neurospora crassa [60,61]. MSUD is reasoned to have evolved for defending against invasion of transposons [62–64]. The connection between MSCI and MSUD is supported by the silencing of unpaired chromosomal fragments in the mouse and worm [65,66].

Each of the three hypotheses for MSCI captures only some specific features consequent to the evolution of sex chromosomes, and hence provides only a partial and proximate explanation for the evolution of MSCI. The ultimate cause of MSCI, of course, must be the degeneration of the Y or W chromosome. Following this line of reasoning, we propose yet another hypothesis that we call “the drive hypothesis” for the evolution of MSCI: there is a constant requirement for silencing sex-linked genes including potential *sex-ratio* distorters during meiosis because of an intrinsic conflict over the sex ratio within a genome accompanying sex chromosome evolution.

We believe that the drive hypothesis provides mechanistically superior explanations to the other hypotheses for the evolution of MSCI for the following specific observations: (1) There is a predominant pattern of generating testis-specific genes through retrotransposition between the X chromosomes and autosomes [67–70]. Some of the retrotransposed sequences might be involved in creating new distorters and suppressors. (2) MSCI in the worm and mouse is dependent on a putative RNA-dependent RNA polymerase, suggesting the involvement of an RNAi-like mechanism [71]. The *Dox*/*Nmy* case provides a strong mechanistic connection between meiotic drive and the evolution of MSCI. Admittedly, it is not an easy task to test empirically and discriminate among the hypotheses described above. Although the drive hypothesis emphasizes the importance of meiotic drive in the evolution of MSCI, other sex-chromosome–specific features such as suppressed recombination, degeneration, and depletion of sex-specific genes could well be different facets of the same evolutionary process. These features may share biochemical components and have reinforced each other over evolutionary time.

In addition to MSCI, achiasmatic meiosis is another evolutionary oddity that might also be rooted in sex chromosome evolution. An achiasmatic meiosis has no crossovers between homologs, even of autosomes. Between 20 and 30 independent evolutionary occurrences of achiasmatic meiosis have been recorded, and all of them are observed in the heterogametic sex [72], with one possible exception in Tigriopus californicus [73,74]. Nevertheless, it is reasonable to generalize that achiasmatic meiosis evolves only in the heterogametic sex, as a result of occasional spillover of the molecular machinery that is simultaneously responsible for both suppressing meiotic drive and recombination between the sex chromosomes. This connection between achiasmy and heterogamety was actually presaged by J. B. S. Haldane and J. S. Huxley [75,76], but here we specify meiotic drive as the major evolutionary cause. One would wonder why there are so many *sex-ratio* meiotic drive cases reported in *Drosophila*. Other than the geneticists' proclivity for counting flies in lab, does achiasmatic male meiosis in this genus also contribute to the abundance of meiotic drive systems?

### Variations on a Theme: Meiotic Drive in Females

Meiotic drive in ZW females is mechanistically different from that in XY males. For XY males, *sex-ratio* meiotic drive can be achieved either by loss of a sex chromosome during meiosis (meiotic drive sensu stricto) or by abnormal postmeiotic development (gametic drive or meiotic drive sensu lato, as used throughout this article). To prevent gametic drive, stringent control of X-linked genes must be achieved through means such as MSCI. However, in ZW females, only one of the four haploid products will eventually end up in the functional egg during oogenesis and meiotic drive sensu stricto would be an easier means to achieve biased transmission than gametic drive.

For the ZW species, centromere structure and centromere-binding proteins may play an important role in meiotic drive (“centromere drive”) [77], and there may be no particular need for evolving MSCI as a defense against gametic drive, although in principle, occasional female gametic drive may still evolve such as a polar body distorter that can kill an egg. Indeed, there is a general lack of cytological observation of MSCI in the ZW females [57,78], with seemingly one proved exception in the caddis fly Glyphotaelius pelludidus [79]. Consistent with this observation, there are only two independent origins of ZW achiasmy [72]. Another explanation for the absence of Z allocycly might be due to a lack of selection pressure to evolve *sex-ratio* distorters on the Z chromosome, because male-biased *sex-ratio* distorter cannot persist long in a population [13], therefore it might have left little trace of impact on genomic evolution prior to its disappearance. The question of whether the Z chromosome lacks MSCI deserves special attention because its general absence would be inconsistent with the SAXI or MSUD hypotheses.

## Materials and Methods

### Fly stocks.

The D. simulans stocks are: (1) *y w^am^ v^2^ f^66^* from the Tucson *Drosophila* Stock Center; (2) *w; e* and simB (*w; nt; III*) [80]; (3) *C(1)RM y w*/*lz^s^* and *Ubx/D* from J. Coyne; (4) *SSR*12-2-7 (*w*; *nt*; *nmy*) [24]; and (5) Paris *sex-ratio* X chromosome *SR6* and its standard *ST8* as described previously [81]. The *SR6* and *ST8* X chromosomes are maintained by backcrossing males to females of the stock *C(1)RM y w*/*lz^s^* every generation. The D. sechellia stock 3588 is from A. Clark [23].

The D. mauriatiana × D. simulans introgression lines have been described before [18,80]. The following lines were used in this study: heterozygous introgression lines *P*40–46 *nmy*, *P*38–11 *nmy*, *P*38H77 *Nmy*, *P*40L12 *nmy*, and *P*40B13 *Nmy*, all having the genotype *w; nt; P*/ *III* (*Nmy*) where *P* represents the various semi-dominant *P*-element transgenes *P*[*w* ^+^] marking the introgressed D. mauritiana material nearby, and *III* (*Nmy*) represents the third chromosome *III* with *Nmy*. These chromosomes are maintained by backcrossing *P*/*III* males to simB females every generation. The *SSR* (skewed sex ratio) line Q15.3 is from a previous D. simulans × D. sechellia hybridization experiment [23]. Several stocks were constructed for this study: *C(1)RM y w*/*w; nt; III* by backcrossing females of *C(1)RM y w*/*lz^s^* to simB males for >19 generations. *C(1)RM y w*/*w dox*; *nt; nmy* was constructed through a scheme described in Figure S5. Another stock, *w*/*Y*[*sech*]; *nt; III*, which is isogenic to simB except that the Y chromosome is from D. sechellia 3588, was constructed through a scheme described in Figure S6. The stock *y w^am^* was constructed from the stocks *w; e* and *y w^am^ v^2^ f^66^*. Another stock, *C(1)RM y w* /*lz^s^ v^2^ f^66^*, was constructed from *C(1)RM y w*/*lz^s^* and *y w^am^ v^2^ f^66^*.

### Fly work.

All flies were reared on cornmeal-molasses-agar medium sprinkled with yeast grains at room temperature (22 ± 1 °C) unless otherwise indicated. The sex ratio of a male was scored by mating this male with three tester virgin females, usually of the stock *w; e*, for 7 d before clearing all adults. The progeny were sexed and counted three times until the 19the day. Sex ratio (*k*) was calculated as percentage of females.

### Molecular biology.

SNPs were discovered by sequencing 500–1,000–bp PCR products from relevant X chromosomes. The primers were designed by targeting the D. melanogaster genome (http://www.flybase.org/), and the virtual PCR products were compared to the D. simulans sequences (http://genome.wustl.edu/tools/blast) for correcting any mismatches within the primers (Table S1). Genotyping was done directly by sequencing. Some other key reagents/kits are: LA Taq long PCR kits (Takara); EZ-Tn5 Insertion Kit for sequencing large DNA fragments (Epicentre); Lambda ZAP II vector for genomic library (Stratagene); TRIZOL Reagent for RNA isolation, SuperScript II Reverse Transcriptase and 3′ or 5′–RACE kits (Invitrogen).

### Cytology.

Light microscopy and TEM procedures have been described previously [24].

## Supporting Information

Figure S1 How Was the *sex-ratio* Distorter Discovered? Q15.3 is one of the original *SSR* (skewed sex ratio) lines reported [23]. This line had been losing the strength of *sex-ratio* distortion by August 2002 (*k* = 0.595; *n* = 10). One male, however, sired an all-female brood (109 F_1_). In another test, 150 eggs sired by a single male developed into 126 female adults. All-female broods are very rare for *nmy* stocks. We were curious whether some new *sex-ratio* mutations had been invading the Q15.3 stock. We crossed *P*40–36 males (*w*; *P*[*w* ^+^] *nmy*/*Nmy*) en masse to females from the latter all-female brood (A, *G2*). The stock *P*40–36 *nmy* was constructed during the positional cloning of *nmy* [24]. A total of 238 males at *G3* were singly mated to three *w; e* females to score sex ratio, and the genotypes of these males can be readily grouped into 12 phenotypic classes out of 16 possible genotypes (A). Twenty-two males (marked with *) expressed *sex-ratio* (*k* ± SEM = 0.986 ± 0.004, which is significantly stronger than a typical *nmy* male tested at the room temperature with *k* ranging from 0.754 to 0.947). Particularly, nine out of the 22 males sired all-female broods. This observation suggests that the distorter on the X chromosome from Q15.3 is a hypermorph or there are other new enhancers for *sex-ratio*. However, the observed numbers of genotypes 1 and 4, and that of genotypes 9 and 12, are significantly smaller than expected (3/55 versus 27.5/55 from the genotypes 1–4, G-test, *p* < 0.001; 19/59 versus*.* 29.5/59 from the genotypes 9–12, *p* < 0.01, regardless of the magnitude of the cross-over rate, s, between *e* and *nmy*). One explanation is that the distorter on the X chromosome from *w; e* might have lost its wild-type function. This explanation is supported by a tighter correlation of the *sex-ratio* phenotype with the X chromosome from Q15.3 (*w* ^+^) than that from *w; e* (*w*) (19/59 from genotypes 9–12 versus 3/55 from genotypes 1–4, *p* = 0.003). Assuming the crossover rate between *w* and a *sex-ratio* distorter (*D*) is r, and there is a loss-of-function mutation (*d*) from the stock *w; e*, the current observation can be adequately explained as detailed in (B). Note that the genotypes 5–8 and 13–16 in (A) are not informative. From the genotypes 1.1–4.2, r and s can be estimated as 10.98% and 32.53%, respectively. From genotypes 9.1–12.2, these two estimates are 37.33% and 22.79%, respectively. The values of s are consistent with the position of *nmy*, which is roughly in the middle of *Ubx–pe* interval [24], while these two latter mutations are 23.8 cM and 44.9 cM distal from *e*, respectively. *D* could be anywhere on the X proximal to *cv* (1–19.3; 11.1 cM proximal to *w*) [82].
(475 KB PDF)

Figure S2 Testing the X Chromosome in the *nmy* Background This scheme was used to verify that the X from the stock *w; e* has a loss-of-function mutation for the *sex-ratio* distorter. In *G1,* the # symbol represents either *P38–11 nmy* or *P38H77 Nmy* and the X symbol represents the X chromosome from any stocks including the following: *w; nt; III*, *w; e*, and *y w^am^ v^2^ f^66^*. In *G3*, the X chromosomes were tested either in unsuppressing (*nmy/nmy*) or suppressing (*nmy/Nmy*) background. In *nmy/nmy* background, the X chromosomes from *w; nt; III* (simB) and *y w^am^ v^2^ f^66^* expressed strong *sex-ratio*, whereas the X from *w; e* only expressed very weak *sex-ratio* (*t*-test, * *p* < 0.05; *** *p* < 0.001).
(233 KB PDF)

Figure S3 Phylogenetic Analysis of the 360-bp Repeats among the D. melanogaster Sibling Species The phylogenetic tree was constructed using MEGA 3.1 with Kimura 2-parameter and Neighbor-Joining algorithm, and was evaluated by bootstrap [83]. These repeats are defined in GenBank accessions EF596890–EF596893, and AE014298.4 (937..1245, 1246..1604, 1605..1963, 1964..2322, 2323..2681, 2682..3040, 3041..3399, 3400..3758). The eight repeats from D. melanogaster form a single cluster. However, all the repeats from the other three species are intermingled, suggesting a history of gene conversion and/or incomplete lineage sorting.
(215 KB PDF)

Figure S4 No Transcripts from *CG32702d* Were Detected The primers CG32702seqF26 and CG32702seqR26, which flank both the divergent base and intron 13 (Figure 4), were used to amplify a 774-bp fragment from genomic DNA (gDNA) and a 706-bp from cDNA. The PCR products were cut with *Bsa*WI that has a restriction site at position 463 on the *CG32702* allele but not on the *CG32702d* allele. No cDNA band of 706 bp was detected, suggesting there is no transcription across intron 13 in *CG32702d*. M indicates DNA size marker.
(262 KB PDF)

Figure S5 Construction of a Double-Mutant Stock for *dox* and *nmy*, *C(1)RM y w Dox*/*w dox; nt; nmy*
In *G2*, *P*40L12 *nmy* can have recombination with *III* (*Nmy)* to produce P40L12 *Nmy*, which in turn can have recombinations with *nmy* at *G4*. This rare possibility was excluded after *G5* by Southern blots.
(224 KB PDF)

Figure S6 Introgression of the Y Chromosome of D. sechellia into Pure D. simulans Background (A) The introgression scheme used here is different from previous ones [84,85]. The D. sechellia Y chromosome (*Y*[*sech*]) was substituted into a pure D. simulans background that is isogenic to simB. *Y*[*sech*] comes from the line 3588 of D. sechellia. The symbol # represents possible hybrid chromosome between D. simulans and D. sechellia. *P*38–11 provides a dominant marker.
(B) A schematic of the Y chromosome (modified from [86]). To verify that the Y introgression line *D. simulans Y*[*sech*] is authentic, we developed a 3.4-kb Y-specific probe (*Y5g*) that was used to fingerprint the Y chromosomes from D. melanogaster, *D. simulans*, *D. mauritiana*, and D. sechellia. D. melanogaster sequences from five Y-specific genes (three from *kl5*, *kl3*, and *kl2*, and two possibly from *ks1* and *ks2*) were used to design PCR primers [87,88]. The five Y-specific PCR products from D. simulans are as follows (size in bp): kl5F-kl5R (670), kl2F-kl2R (930), kl3F-kl3R (879), ks1F-ks1R (575), and ks2F-ks2R4 (351). A PCR trick was used to join two DNA fragments as follows: In the first PCR, a chimera primer, which consists of the reverse primer of the first fragment and the antisense forward primer of the second fragment, was used to make an anchored fragment. In the second PCR, the forward primer of the first DNA and the reverse primer of the second DNA were used to amplify a product consisting of these two DNA fragments, with a mix of the anchored fragment and the second DNA as template. *Y5g* was made by joining the above five fragments sequentially, and was confirmed by sequencing. Southern blots using *Y5g* show species-specific pattern for the Y-chromosome (Figure 7A).
(304 KB PDF)

Table S1 Primers Used for SNP Markers (91 KB DOC)

Text S1 The Gene *CG32702* and Its Transcript in D. simulans
(35 KB DOC)

Text S2 The Coding Potential of *Dox* and *MDox*
(46 KB DOC)

Text S3 Some Primers Used in This Study (26 KB DOC)

### Accession Numbers

All sequences have been deposited in the GenBank database (http://www.ncbi.nlm.nih.gov/Genbank/index.html) and have been assigned the accession numbers EF596886-EF596899.

## Abbreviations

aa: amino acids
DAPI: 4',6-diamidino-2-phenylindole
dsRNA: double-stranded RNA
IR: inverted repeats
MSCI: meiotic sex chromosome silencing
MSUD: meiotic silencing of unpaired DNA
ORF: open reading frame
RACE: rapid amplification of cDNA ends
RT-PCR: reverse-transcription PCR
siRNA: small interfering RNA
TEM: transmission electron microscopy
TGS: transcriptional gene silencing
