# Supplementary material for: A sex-ratio Meiotic Drive System in Drosophila simulans. II: An X-linked Distorter
Source: PLoS Biol. 2007 Nov 6;5(11):e293. doi: 10.1371/journal.pbio.0050293 (PMC2062476; doi:10.1371/journal.pbio.0050293)
Supplement: Supplementary file 1 [file pbio.0050293.sg001.pdf]

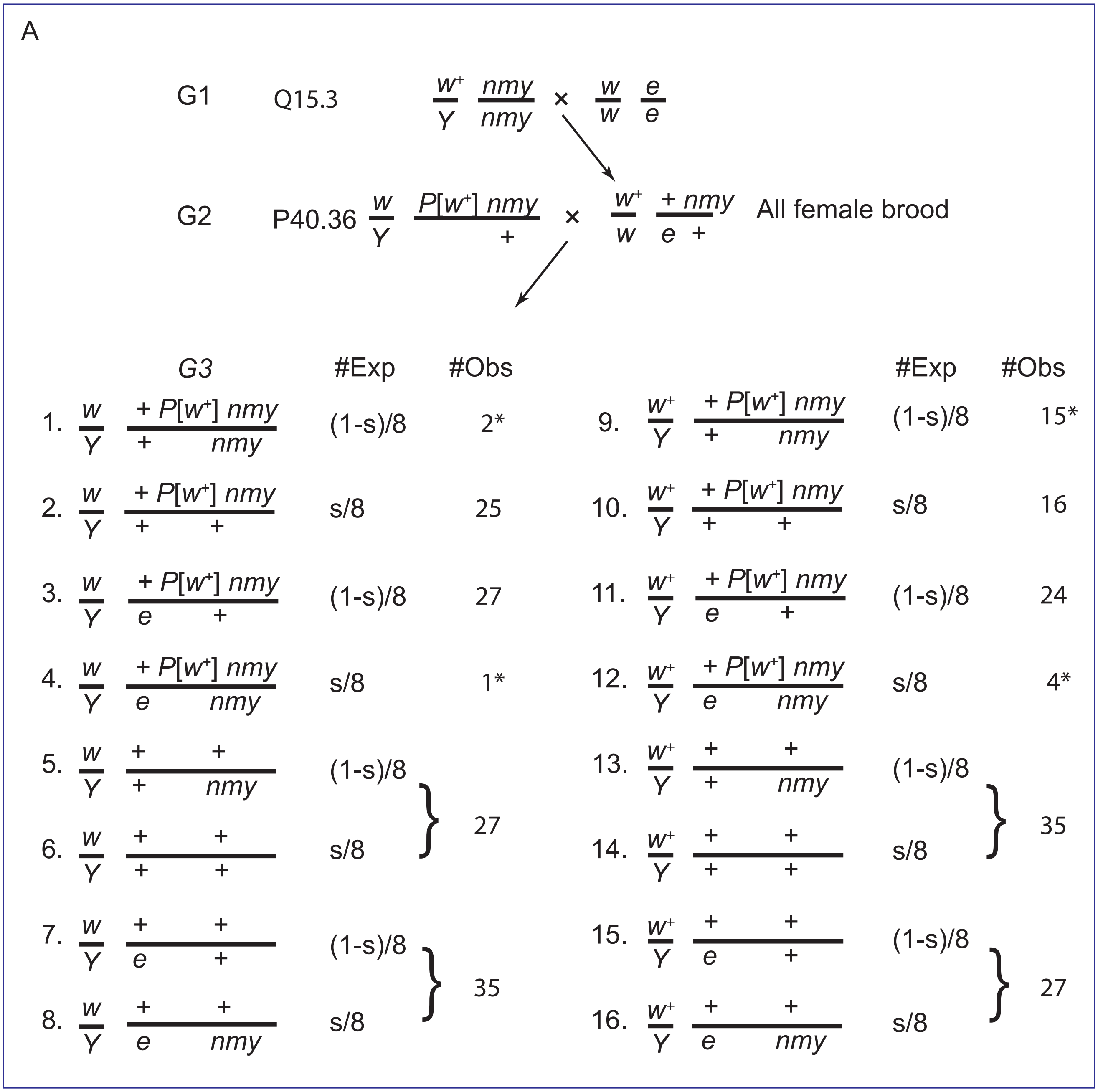

* Expressing *sex-ratio*

Figure S1

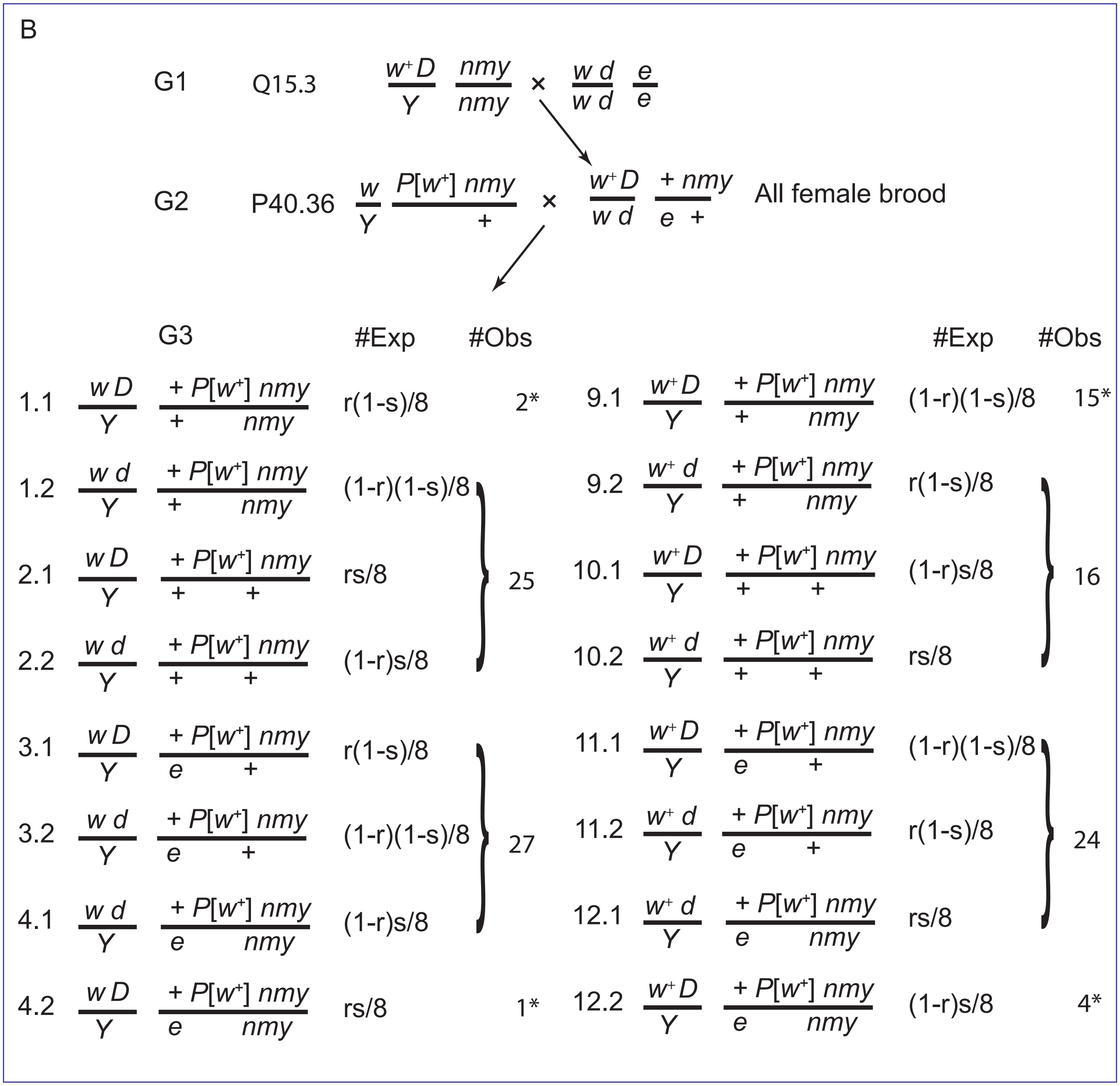

* Expressing *sex-ratio*

Figure S1
