# Supplementary material for: A sex-ratio Meiotic Drive System in Drosophila simulans. II: An X-linked Distorter
Source: PLoS Biol. 2007 Nov 6;5(11):e293. doi: 10.1371/journal.pbio.0050293 (PMC2062476; doi:10.1371/journal.pbio.0050293)
Supplement: Supplementary file 2 [file pbio.0050293.sg002.pdf]

G1 *w Dox/Y; nt; P*40L12 *nmy/ III* × C(1)RM *y w/Y; nt; III*

w Dox/*Y*; *nt; #/ III* × 𝕏*; +; +* (or 𝕏*; +; +*)

G2 C(1)RM *y w/Y; nt*; *P*40L12 *nmy/ III* × 𝕏*/Y; nt/+*; #/ + (or 𝕏*/Y; nt*; #/ +)

G3	Sex ratio (k)		
	w Dox; nt; III (simB)	*w; e*	*y* w^{am} v^2 f^{66}
𝕏*/Y; nt; P40L12 nmy/ P38-11 nmy*	0.816 ± 0.009 (32)	0.538 ± 0.013 (21)	0.954 ± 0.007 (14)
𝕏*/Y; nt; P40L12 nmy/ P38H77 Nmy*	0.505 ± 0.007 (26)	0.500 ± 0.008 (27)	0.524 ± 0.007 (6)
	***	*	***

Figure S2
