# Supplementary material for: A sex-ratio Meiotic Drive System in Drosophila simulans. II: An X-linked Distorter
Source: PLoS Biol. 2007 Nov 6;5(11):e293. doi: 10.1371/journal.pbio.0050293 (PMC2062476; doi:10.1371/journal.pbio.0050293)
Supplement: Supplementary file 3 [file pbio.0050293.sg003.pdf]

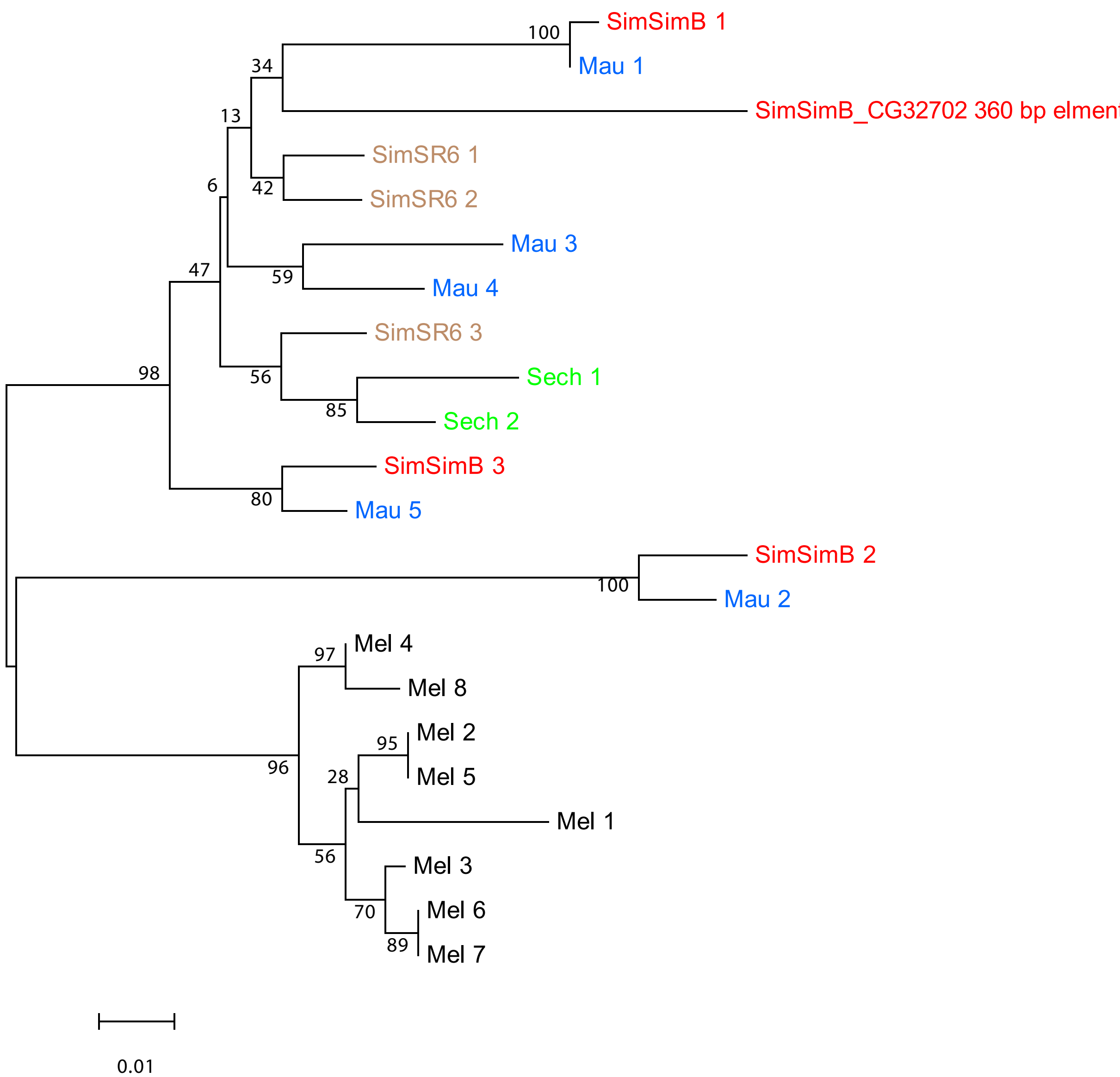
SimSimB 1
Mau 1
SimSimB_CG32702 360 bp elment
SimSR6 1
SimSR6 2
Mau 3
Mau 4
SimSR6 3
Sech 1
Sech 2
SimSimB 3
Mau 5
SimSimB 2
Mau 2
Mel 4
Mel 8
Mel 2
Mel 5
Mel 1
Mel 3
Mel 6
Mel 7
100
34
13
42
6
59
47
98
56
85
80
100
97
95
28
96
56
70
89
0.01

Figure S3
