# Supplementary material for: A sex-ratio Meiotic Drive System in Drosophila simulans. II: An X-linked Distorter
Source: PLoS Biol. 2007 Nov 6;5(11):e293. doi: 10.1371/journal.pbio.0050293 (PMC2062476; doi:10.1371/journal.pbio.0050293)
Supplement: Supplementary file 5 [file pbio.0050293.sg005.pdf]

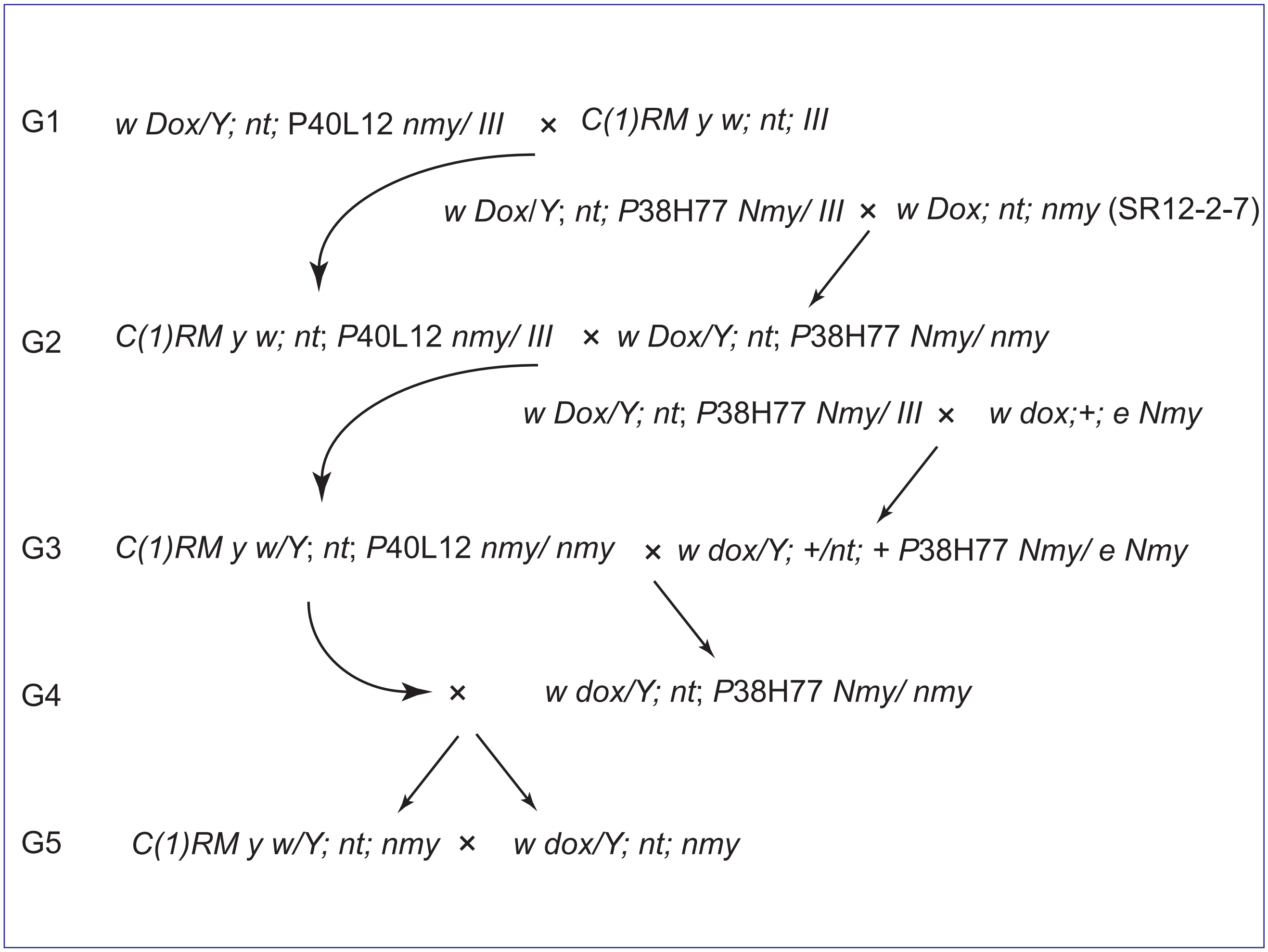

G1
w Dox/Y; nt; P40L12 nmy/ III × C(1)RM y w; nt; III
w Dox/Y; nt; P38H77 Nmy/ III × w Dox; nt; nmy (SR12-2-7)
G2
C(1)RM y w; nt; P40L12 nmy/ III × w Dox/Y; nt; P38H77 Nmy/ nmy
w Dox/Y; nt; P38H77 Nmy/ III × w dox;+; e Nmy
G3
C(1)RM y w/Y; nt; P40L12 nmy/ nmy × w dox/Y; +/nt; + P38H77 Nmy/ e Nmy
G4
× w dox/Y; nt; P38H77 Nmy/ nmy
G5
C(1)RM y w/Y; nt; nmy × w dox/Y; nt; nmy

Figure S5
