# Supplementary material for: A sex-ratio Meiotic Drive System in Drosophila simulans. II: An X-linked Distorter
Source: PLoS Biol. 2007 Nov 6;5(11):e293. doi: 10.1371/journal.pbio.0050293 (PMC2062476; doi:10.1371/journal.pbio.0050293)
Supplement: Supplementary file 6 [file pbio.0050293.sg006.pdf]

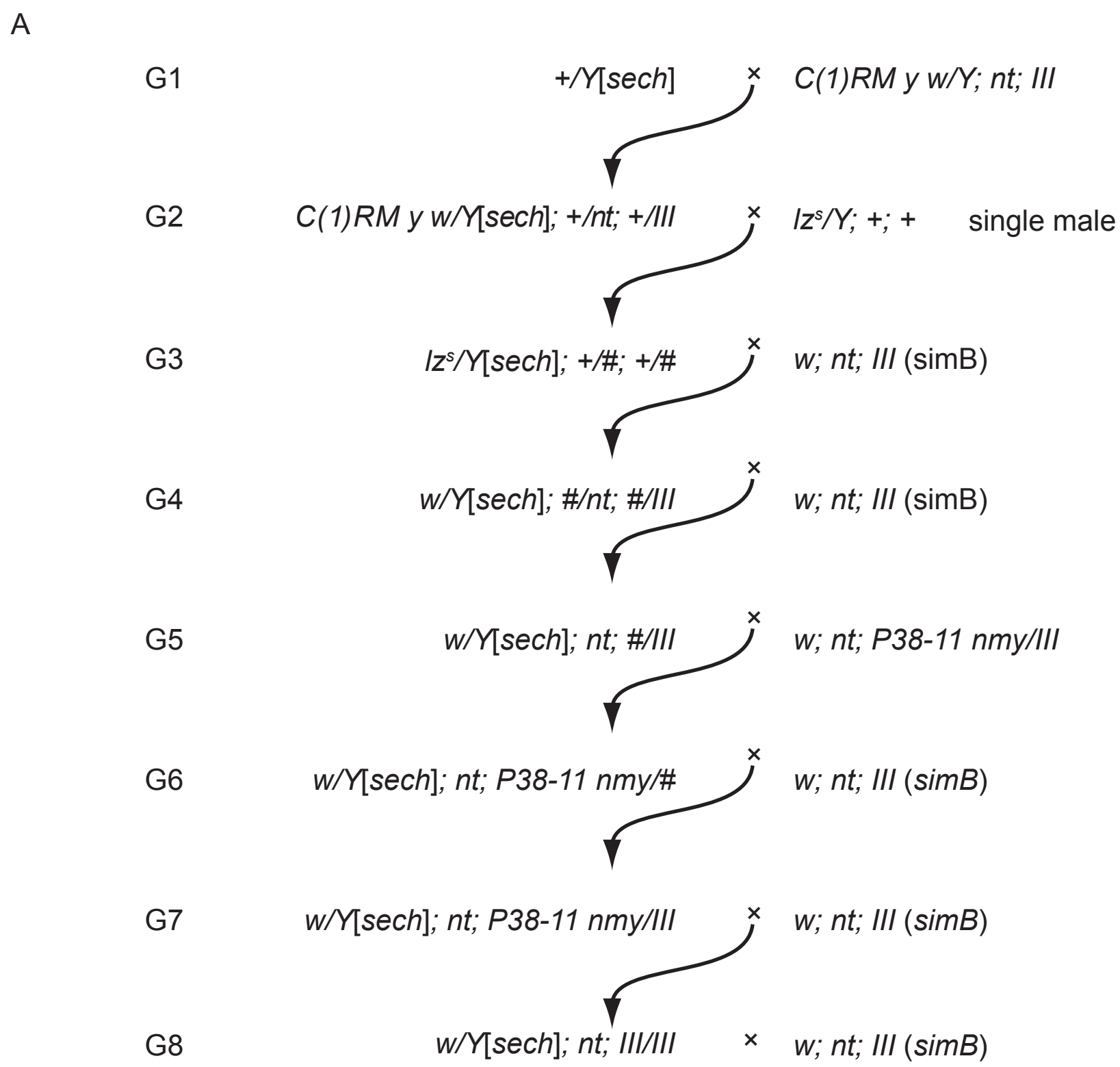
A
G1 +/Y[sech] × C(1)RM y w/Y; nt; III
G2 C(1)RM y w/Y[sech]; +/nt; +/III × lz^s/Y; +; + single male
G3 lz^s/Y[sech]; +/#; +/# × w; nt; III (simB)
G4 w/Y[sech]; #/nt; #/III × w; nt; III (simB)
G5 w/Y[sech]; nt; #/III × w; nt; P38-11 nmy/III
G6 w/Y[sech]; nt; P38-11 nmy/# × w; nt; III (simB)
G7 w/Y[sech]; nt; P38-11 nmy/III × w; nt; III (simB)
G8 w/Y[sech]; nt; III/III × w; nt; III (simB)

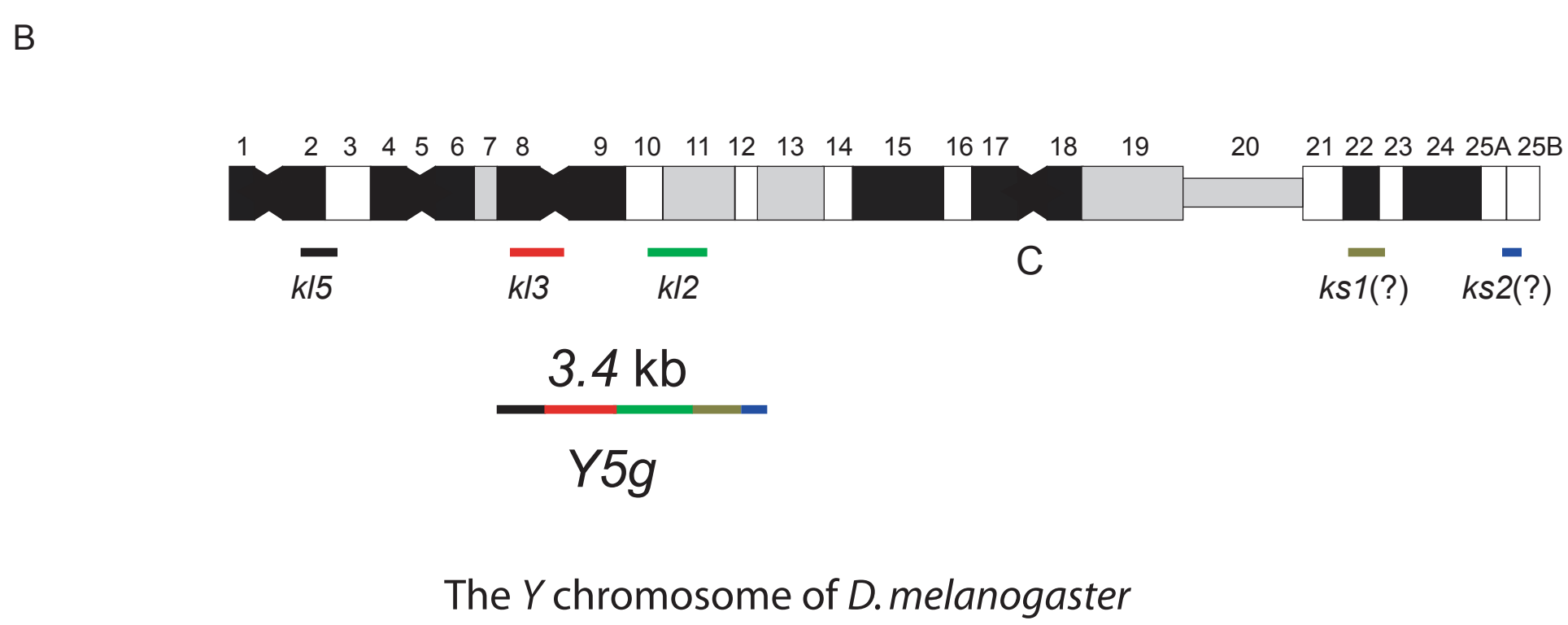
B
1 2 3 4 5 6 7 8 9 10 11 12 13 14 15 16 17 18 19 20 21 22 23 24 25A 25B
kl5
kl3
kl2
C
ks1(?)
ks2(?)
3.4 kb
Y5g
The Y chromosome of D. melanogaster

Figure S6
