# Supplementary material for: A sex-ratio Meiotic Drive System in Drosophila simulans. II: An X-linked Distorter
Source: PLoS Biol. 2007 Nov 6;5(11):e293. doi: 10.1371/journal.pbio.0050293 (PMC2062476; doi:10.1371/journal.pbio.0050293)
Supplement: Supplementary file 7 [file pbio.0050293.sd001.doc]

**Text S1:** The Gene *CG32702* and its Transcript in *D. simulans*

A 507 bp probe (nmyIRF - nmyR3004) from the *Nmy* region was used to screen a *D. simulans* phage genome library. One positive clone contains an 8493 bp insert that does not match to either the *Nmy* or *Dox* region but to the gene *CG32702*. We therefore used two pairs of primers (sim_cg32702F1 and sim_cg32702R1, sim_cg32702F2 and sim_cg32702R2) to extend the coverage and a total of 18,733 bp of sequence was obtained (Note: A small piece of ~245 bp near the primer sim_cg32702 R2 was not sequenced.) The full length cDNA of *CG32702* was also obtained by using 5’- and 3’-RACE and RT-PCR with numerous primers. It has predicted molecular functions of calcium ion binding and receptor activity, but lacks any experimental evidence. A full-length mRNA of 3331 bp (*AY084200*) from *D. melanogaster* is found in database, largely corresponding to the last 4 predicted exons (exons 10-12). However, exon 2 of *AY084200* (corresponding to exon 11 of *CG32702*) has an alternative splicing with its donor site 5 nucleotides prior to that in *CG32702*. *AY084200* thus has an earlier termination of translation. No evidence shows the existence of an *AY084200*-like transcript in *D. simulans*.
