# Supplementary material for: A sex-ratio Meiotic Drive System in Drosophila simulans. II: An X-linked Distorter
Source: PLoS Biol. 2007 Nov 6;5(11):e293. doi: 10.1371/journal.pbio.0050293 (PMC2062476; doi:10.1371/journal.pbio.0050293)
Supplement: Supplementary file 8 [file pbio.0050293.sd002.doc]

**Text S2.** The Coding Potential of *Dox* and *MDox*

The coding potential of both *Dox* and *MDox* appears to be very limited (Figure 4). We evaluate the 9 and 8 potential ORFs > 100 bp in all three reading frames of *Dox* and *MDox*, respectively, by comparing the four nucleotides prior to AUG in each ORF to the consensus translation start sites used by *Drosophila* (Cavener and Cavener, 1993. In: Maroni G, ed. An Atlas of *Drosophila* Genes: Sequences and Molecular Features. N.Y.: Oxford University Press, Inc. pp. 359-377). Each ORF is ranked by the index $j=N_{i}\underset{-4}{}$ where $N_{j}=$ one of A, C, G or T, and $P_{i,j}$ is the nucleotide frequency in each of the four translation start sites prior to AUG. The ORF across the exon III region (bracketed) is ranked first among the 9 *Dox* ORFs shown in Figure 4. Similarly, the corresponding ORF is ranked second among the 8 *MDox* ORFs. These two ORFs appear to be strong candidates that are actually translated. The real protein product, if any, has to be determined through *in vitro* and *in vivo* translation experiments. However, since non-AUG translation start sites, such as CUG, have been reported in *Drosophila* (Boyd and Thummel, 1993. *Proc. Natl. Acad. Sci*. 90: 9164-9167), unexpected protein products cannot be excluded without experiment data.
