# Supplementary material for: A sex-ratio Meiotic Drive System in Drosophila simulans. II: An X-linked Distorter
Source: PLoS Biol. 2007 Nov 6;5(11):e293. doi: 10.1371/journal.pbio.0050293 (PMC2062476; doi:10.1371/journal.pbio.0050293)
Supplement: Supplementary file 9 [file pbio.0050293.sd003.doc]

**Text S3:** Some Primers Used in this Study

```
5Dox14k.F1 		gtgcgtatGagtaacccacagtttggtctg
5Dox14k.R1		aaaaacttagGatcggacgggactcGaaat

5Dox14k.F3		TAATATGCCCACCAAGAGTCTACAAATACACG
5Dox14k.R5		AGCCGATAAGTATGAATTTCGTTTCGATTCAA

5Dox14k.F6		AAGTCGAGTTCTCAATAATCCCTGTCCACTTA
5Dox14k.R3		CCACAATGCGCTTATTTAGAATGTGTGTTC

DoxF2			cagtttctatcgaaaactagagtccaaaggctacg
DoxR2			agaaggttccttagagtttggcgactaccatgtgc

3Dox31k.F1		gccctatttAgagaGcttcacctttatgtttct
3Dox31k.R1		gcaattagcatttaattgtgcgaaccaaactat

DoxF4			AAGCAGTTCCCAAAGAAAGAGAGCAGAGCAACT
DoxR4			CCAGCTCAAAACCACAGGGAGACACTGTACATA

sim_cg32702F1		gtactgccatcgttgaCcaaaacaaattcc
sim_cg32702R1		ggatgcagtcttagtcccaaAgtagattgc

sim_cg32702F2		Tggtaaatggttaccacaatagcgtccaat
sim_cg32702R2		aatatgacactttcacCtggacctgggaCg

sim_cg32702R3		ATCTTCTACTCCGCAATGGAAACGATGAAG
sim_cg32702F3		CTTGTAGTACTTTGGATTGTCCTCGCCACA

kl5F				tagatgtgcacagtcgtgat
kl5R				tcgctaattaaggcaaagtc

kl2F				gaacctcctcgtggaatcaa
kl2R				taatggcacccttccttcag

kl3F				agttagtggcaatgaacggg
kl3R				acttccccaagcatgtaacg

ks1F				tgcacaaaatgcccttgata
ks1R				aatttcgaggtcgcattcac

ks2F				gcacaggcattcattttcct
ks2R4			cctgcatattcgcctgaaat

nmyIRF			ATTACTGCCACTCTCCGACG
nmyR3004			TACAACACGTCGATACGATTC

RpL32f1			ccttccagcttcaagatgacc
RpL32r2			tcaagaatcttaagcttactcg

cg32702seqR26		ACACAGCTTCTCGGATGACC
cg32702seqF26		GGAGGTCTGGCCATTGAATA
```
