# Supplementary material for: A sex-ratio Meiotic Drive System in Drosophila simulans. II: An X-linked Distorter
Source: PLoS Biol. 2007 Nov 6;5(11):e293. doi: 10.1371/journal.pbio.0050293 (PMC2062476; doi:10.1371/journal.pbio.0050293)
Supplement: Supplementary file 10 [file pbio.0050293.st001.doc]

**Table S1**. Primers Used for SNP Markers

| **Name (Cyto. location)** | **Forward Primer (.F)** | **Reverse Primer (.R)** |
| --- | --- | --- |
| *CG14622* (1F2-3) | GCCTTCCACCACACAAATCT | GCCGCTCCAGAAGCACTACA |
| *CG3585* (5E1-3) | TGCCATGTGGTAAATGGGTA | GTGACCCTGTCCTGGAATCT |
| *CG1643* (7B1) | tccattagcctccgattgac | gcctcaccattcacttctcc |
| *CG12654* (8D10) | aatttgcggcctttaccttg | gggcagtacacgttcaacaa |
| *CG15316* (8E1-4) | CCCGTCGACGTATCTTTTGT | TTTTCTGCGCTTTCCACTTT |
| *5Dox_III* (8F1) | GGCGGTCAAGTCTATGGAAA | CCAAAGTCTCGTGCTTCTCC |
| *G0232* (8F2-6) | GATTTCCCAGCCAAATTTCC | GGTTGGTTTTCTTGGCAGGT |
| *CG3106* (8F6) | TCAACAACTTGGAGGAGTGG | TTAAGGGGGCTCGATATGA |
| *nej* (8F7-9) | TTGATTGTACCCCTTAAATGTT | GGCGACACCGTATTCCTTTA |
| *CG15245* (9A2) | GGTGATCCCTCATTTTTCCG | GTAAATTCCCCTGCGCTTTC |
| *CG1799* (9E1-2) | CCAATAAGCAGCTTCTTGTC | CCGGCATCAATGAGATTTTT |
| *CG1582* (9F8) | acggattcgatcagttctgg | ccgactggaaaacaaggtgt |
| *CG4645* (11D1-2) | gtgggccctcttcaagtaca | gcactcaggattcccacgat |
| *CG1517* (12E6-7) | cagatgaccattttgcatcg | gcagcaacttaatgtccaga |
| *CG32575* (14C4) | ATGGTCGATCCCACAATGTT | GGTGAGCAGTGGTCGGTACT |
| *CG5675* (16B7) | TCAGTCCCACGGGCTCTAT | CAGATCGCCAATGTCGGCTA |
| *CG6659* (17C7-D1) | GACAAAGCCGACATTGACCT | TGCATCGGAAGGATCATACA |
| *CG7440* (18A3) | CGGAGCGCTGGAACTGGTAG | TACAAGATGGTCGGCACTCA |
